# Human factors in digital manufacturing technology adoption: a workforce perspective

**DOI:** 10.1007/s00170-025-16524-5

**Published:** 2025-10-07

**Authors:** Anne-Marie Oostveen, Iveta Eimontaite, Sarah Fletcher

**Affiliations:** Centre for Robotics and Assembly, Faculty of Engineering and Applied Sciences (FEAS), Cranfield University, Cranfield, MK43 0AL UK

**Keywords:** Digital manufacturing technologies, Human factors, Technology acceptance, Workforce adaptation, Industry 4.0, User and non-users

## Abstract

The UK is the twelfth-largest manufacturing nation globally, yet its adoption of digital manufacturing technologies (DMTs) lags behind other European countries. In an era where industrial automation and digital transformation are essential for maintaining competitiveness, understanding the human factors influencing the acceptance and implementation of these technologies is critical. This study examines the perceptions of 313 UK manufacturing employees regarding the usefulness, ease of use, and workplace impact of DMTs. Findings indicate that while employees recognise the potential benefits of DMTs such as increased productivity, improved product quality, and enhanced competitiveness, concerns remain regarding ease of use, workforce upskilling, and physical interaction with new technologies. Notably, employees with lower educational qualifications expressed greater scepticism about the applicability of DMTs. Furthermore, those working in companies that had already implemented digital technologies reported more positive perceptions compared to non-users, emphasising the role of experience in shaping attitudes. The study highlights the need for targeted training and change management strategies to facilitate smoother workforce adaptation to digital advancements. These findings provide insights for policymakers, industry leaders, and system designers aiming to integrate human-centric approaches in the transition to Industry 4.0 and beyond.

## Introduction

In the last decade, several parallel technological developments have enabled industries worldwide to embark on large-scale digital transformations aimed at boosting productivity, enhancing quality, and sustaining competitiveness. In line with this global movement, the UK manufacturing sector has increasingly invested in high-end ‘digital manufacturing technologies’ (DMTs) to modernise production systems and support export-driven growth. DMTs comprise a wide-ranging set of intelligent and interconnected tools including robotics, sensor technologies, Artificial Intelligence and analytics, distributed networking technologies, additive manufacturing, simulation, Virtual and Augmented Reality, the Internet of Things, and cloud computing. Those technologies hold the promise of enhancing operations, such as design, planning, scheduling, monitoring, data management, decision support, commerce, and logistics. Szalavetz [1] points out that the digital transformation of manufacturing not only strengthens production quality and efficiency but also enhances a company's flexibility, agility, and responsiveness to market changes. With the increasing adoption of DMTs in industries worldwide, UK manufacturing is driven towards the rising deployment of digital technologies to sustain global competitiveness.

Despite these opportunities, the UK manufacturing sector faces significant challenges. While manufacturing contributes only 10% to the UK economy and 9% to its total employment (2.6 million jobs), it accounts for a disproportionate share (45%) of total exports [2]. The UK is currently the world's twelfth-largest manufacturing nation by output, having dropped from ninth place in 2019. The United States is the UK’s largest export market reaching £61.8 billion, while European markets remain important, as six of the UK’s top ten export markets are within the EU, collectively worth approximately £150 billion [2]. The UK’s frictionless trade interconnections with Europe, which have been the norm for decades, is further threatened by Brexit-related trade frictions and market uncertainties [3]. In this climate, DMTs are increasingly viewed as essential investments to mitigate risks and safeguard long-term competitiveness.

One crucial DMT is industrial automation. Yet, despite its potential, the UK lags behind its European and global peers in automation uptake. Compared with other European countries UK adoption could have been faster, particularly amongst Small and Medium Enterprises (SMEs) with a vast majority of 20,000 out of 27,000 SMEs currently operating without robotics [4]. Exacerbated by Covid-19 and Brexit, the UK has faced a surprising decline in manufacturing and industrial productivity [5]. According to an International Federation of Robotics report, the UK is the only G7 country with a robot density below the world's average, with just 111 industrial robots per 10,000 workers in comparison to Germany with 397 units and the Republic of Korea with 1000 units [6]. This presents a significant challenge for UK manufacturers, as the country ranks 24th globally in robot density [7]. In terms of robotics adoption, the UK is now being outpaced by Slovenia, Slovakia, Finland, and Hungary [6]. Several barriers have been proposed in the literature, including skills shortages, high costs, unrealistic performance expectations, and slow return on investment [8, 9]. However, despite these insights, there is a critical lack of robust empirical evidence identifying which specific factors most strongly influence UK manufacturers’ decisions to adopt automation and robotics technologies.

More importantly, while economic, technical, and regulatory factors have received substantial academic attention, the human factors dimension remains underexplored. Bauer, Schlund, and Vocke [10] highlight that workforce readiness, including changes in tasks, equipment, skill requirements, and job security, plays a pivotal role in the success or failure of technological adoption. Nevertheless, most frameworks and readiness assessments for new technologies continue to prioritise technical and economic dimensions, often neglecting employees’ perceptions and acceptance [11, 12]. Given that successful implementation depends on employee buy-in and their ability to adapt to new, mandatory work methods imposed by DMT adoption, the absence of human factors analysis represents a critical research gap.

The study set out in this paper directly addresses this gap by investigating manufacturing employees’ perceptions of DMT adoption in the UK. Specifically, it focuses on identifying human and organisational factors that influence acceptance and perceived barriers. DMT acceptance is vital in the successful adoption of ‘Industry 4.0’ and ‘Industry 5.0’ technologies [13]. Introducing new technologies in a company can change the nature of the work, affect work schedules or staffing levels, influence people’s morale, affect relations with co-workers and supervisors, and improve or worsen levels of accomplishment. The readiness and willingness of the workforce to adopt new technology and change their work methods are crucial to its effective utilisation [14]. By comparing views across different demographics (e.g. age, gender, role, education), organisational sizes, and between employees in firms with and without DMTs, the study provides nuanced insights into the determinants of successful technology integration.

The present research advances existing knowledge in several important ways: it provides empirical evidence on human factors affecting DMT adoption, a dimension sparsely examined in prior work. Furthermore, it examines employees’ perceptions directly, rather than relying solely on managerial or organisational-level perspectives. It also contributes to the development of more comprehensive adoption frameworks, integrating human and technical factors to inform future industrial policy and design strategies. The principal research aim is to identify the perceptions of UK manufacturing employees regarding factors influencing the acceptance and adoption of DMTs. The study addresses the following research questions (RQs):

1. Do manufacturing employees identify the same advantages and barriers to implementing DMTs as described in the literature?
2. Are these opinions influenced by individual characteristics such as age, gender, role, or education?
3. Does organisational size affect employee perceptions and perceived barriers?
4. Do perceptions differ between employees working in organisations that have adopted DMTs and those that have not?

By illuminating employees’ views, this study not only enriches theoretical understanding of digital transformation in manufacturing but also offers actionable insights for managers, engineers, and policymakers seeking to design effective, human-centred strategies for technology adoption. This paper is organised as follows: Section 2 provides an overview of previous research; Section 3 presents the research methodology; Sect. 4 analyses the results from the manufacturing employees’ survey; Section 5 discusses these results, and finally, Section 6 draws conclusions, and considers the limitations of the work and avenues for future research.

## Background

### Barriers to digital transformation

Industrial revolutions are periods of significant transformation to business practices emanating from a pervasive influence of a new technology paradigm. Industry is currently experiencing a fourth major transformation of this magnitude, called ‘Industry 4.0’, due to significant and pervasive advances in technologies that enable digitisation. Widespread integration and interconnection of digital technologies are expected to foster global benefits through significantly improved production capabilities, costs efficiencies, and working conditions [15–17]. In recent years, the emergence of a theoretical vision for ‘Industry 5.0’ has built on the concepts of Industry 4.0 by integrating societal value and human wellbeing as central priorities [18]. Unlike Industry 4.0’s exclusive focus on technological advancement and economic growth, Industry 5.0 seeks to embed human-centric approaches, emphasising worker prosperity and ecological sustainability.

This conceptual evolution signals an important shift: technological implementation should not only focus on economic value but also consider the social, psychological, and ecological impacts on workers and communities. It highlights the necessity to design systems that enable workers to focus on higher-value, creative tasks, supported by automation [18]. Despite these ideals, however, the practical realities of digital transformation reveal substantial barriers, particularly among SMEs, where high upfront investment costs continue to be a prominent barrier to adoption [20–23]. SMEs, constrained by limited financial resources, face greater challenges in justifying large-scale investments without immediate returns [19, 24]. Empirical evidence from UK manufacturing indicates that financial constraints are the most cited impediment to adopting new digital manufacturing technologies [24]. Furthermore, although automation and robotics were initially projected to enable re-shoring of manufacturing to higher-cost countries due to rising global labour costs and cheaper, more capable robots [25], studies show that this shift has largely failed to materialise on a large scale [1, 26]. Instead, there are emerging concerns that widespread adoption of advanced manufacturing technologies could exacerbate job losses, disproportionately affecting less-skilled workers with a lower level of education [27] or those belonging to disadvantaged social classes [28]. Such outcomes challenge the assumption that technological advancement will automatically lead to broad-based societal benefits and highlight the need to consider distributional effects in technology policy and planning.

An additional critical barrier involves the skills gap. The rapid pace of technological change has transformed the demand for workforce skills, requiring employees to continuously update their knowledge and understanding of different technologies and production methods [29]. Sima et al. [28] note that human capital is transitioning. No longer will initial qualifications be sufficient for an entire career; instead, employees will have to continuously acquire new knowledge and skills through periodic training once every few years: “Even people who know how to use and operate technology are required to renew and expand their skills and competencies” (p17) [28]. Education and training systems will play a crucial role in encouraging flexibility and adaptability within the workforce. Traditional education systems need to be abandoned in favour of systems that focus on knowledge beyond what is currently taught such as creative thinking, expertise, and cognitive skills [30] while manufacturing organisations will have to facilitate up-skilling (whereby the existing workforce is trained in carrying out tasks using DMTs such as robots), and re-skilling of their employees (whereby the workers whose jobs will no longer exist are given the new skills that their companies require). Both Industry 4.0 and Industry 5.0 require skills to be continually developed in conjunction with technological developments [31–34]. Moreover, organisational success in digital transformation now depends not only on technical skills but also on so-called "soft" skills, e.g. interpersonal communication, problem-solving, leadership, and adaptability, which are increasingly valued by employers [35]. However, developing these competencies poses particular challenges for SMEs, which often lack the resources to provide tailored training and struggle to attract and retain talent with specialist skill sets [36].

Despite these constraints, SMEs potentially stand to gain the most from DMT adoption. Digital technologies offer smaller production runs, customised products, and increased agility at competitive costs [12, 25, 36–38]. Falling technology costs theoretically create opportunities for smaller firms to integrate DMTs, yet the reality reveals low adoption rates, attributed to ongoing concerns over cost, complexity, and organisational readiness [36]. To address these barriers, initiatives such as the UK’s £20 million Made Smarter Pilot were launched to provide financial support and strategic guidance to SMEs [39]. Although financial support is a crucial step, it is increasingly evident that a purely economic focus overlooks deeper cultural and human-related barriers [31, 40]. A nuanced understanding of these barriers, including financial, technical, organisational, and human dimensions, is essential for designing effective support mechanisms. This comprehensive perspective directly informs our research aim and addresses RQ1 (alignment of perceived barriers with literature) and RQ3 (impact of organisational size on perceptions). By investigating employees’ firsthand experiences, our study offers critical insights for tailoring future policy and industry interventions to encourage wider DMT adoption.

### Human factors

Industry and academia have always focused first and foremost on the technical design of new technologies and, by comparison, neglected human and social factors. However, the potential benefits of DMTs rely on “the right mind set” to realise change and improvements [41]. Although some organisational and financial issues have been researched, others have noted that a traditional lack of consideration of human-centred conditions and culture can be highly detrimental to the success of technology implementation [42–44]. In more recent research wider human factors are being explored, for example a literature review of barriers to DMTs that included workforce skills, anxieties and resistance to change [45], a study of technology acceptance within SMEs [24], and a small survey of operators’ opinions on their requirements for future DMTs [46].

A key human factor that is now more commonly being considered is workforce acceptance. The Unified Theory of Acceptance and Use of Technology (UTAUT) provides a robust framework for understanding these dynamics [47]. According to UTAUT, four determinants influence user acceptance: performance expectancy (perceived improvement in job performance), effort expectancy (ease of use), social influence (perception of important others’ expectations), and facilitating conditions (perceived organisational and technical support). Notably, involving employees in design and implementation processes can significantly reduce resistance and foster positive attitudes toward change [48].

Despite these theoretical advances, tools used to assess organisational readiness often continue to neglect human and social dimensions, focusing heavily on technical infrastructure and economic feasibility [49]. For instance, the UK’s recently developed ‘Publicly Available Specification’ (PAS) for digital readiness only lightly addresses human factors, reflecting an enduring technical bias [50]. Yet, future manufacturing environments will continue to rely on human operators working in tandem with automated systems [51–53]. Thus, understanding and designing for human acceptance is not optional but essential for achieving successful digital transformation.

By explicitly focusing on workers’ perceptions of DMTs, our study addresses a critical gap in the literature. It directly responds to RQ1 and RQ4 by examining whether employees' perceived advantages and barriers align with existing theoretical models and exploring differences between technology users and non-users. Furthermore, by highlighting the centrality of human factors, our study supports more comprehensive and human-centred approaches to technology adoption, contributing to both theory and practice.

### Individual differences influence the perception of technology

Numerous theories have been developed to explain technology acceptance, with one of the most prominent being the classic Technology Acceptance Model (TAM). This framework, introduced by Davis [54], aims to understand and predict how users adopt and utilise technology. TAM identifies two primary factors influencing a user's decision to accept and use technology. The first is Perceived Usefulness (PU), which refers to the degree to which an individual believes that using a particular technology will enhance their performance or fulfil their needs. The second is Perceived Ease of Use (PEOU), defined as the extent to which an individual finds the technology easy to understand and operate. These factors collectively shape users' attitudes toward the technology, which subsequently influence their behavioural intention to use it, ultimately leading to actual usage behaviour. TAM has become a widely adopted model in studies focusing on technology adoption and innovation. Although TAM has been proven to have valid and reliable constructs, a meta-analysis of the literature identified that the theory does not sufficiently take into account external variables such as age, gender, education, and prior experience that have been found to directly and indirectly influence people’s perceptions and usage behaviour [55]. This is why in our study we do address the variables described below.

#### Age

Age has traditionally been associated with slower learning curves, higher error rates, and greater resistance to changing established routines [56–59]. However, emerging research suggests that these assumptions, rooted in outdated stereotypes from the 1980 s, are overly simplistic [56]. Broady et al. [56] argue that differences in attitudes toward technology among older and younger workers are often exaggerated, and similar factors shape attitudes across age groups. This insight highlights the importance of avoiding generalisations and considering contextual and individual factors instead.

#### Gender

Gender is another variable that was initially absent from TAM [54]. While findings are mixed, studies such as those by Gefen and Straub [60] suggest that gender moderates the influence of PU and PEOU, with men being more influenced by usefulness and women by ease of use. Conversely, other research has found no significant gender differences [61]. Nevertheless, given the increasing participation of women in technology-oriented roles, it is essential to account for potential gender-related differences when designing and implementing new systems [47]. Understanding these dynamics can facilitate more inclusive change management strategies and help tailor communication and training efforts.

#### Education

Educational level significantly influences technology acceptance. Higher educational attainment is positively associated with greater perceived usefulness and ease of use, as well as stronger attitudes toward adoption [55]. Education not only provides a foundation for understanding new technologies but also reduces anxiety and fosters a more positive disposition toward continuous learning. As such, considering education as a moderating factor is crucial when evaluating organisational readiness and designing training programmes.

#### Prior experience (users vs. non-users)

Although previous studies have identified that users and non-users reveal different perceptions, beliefs, and attitudes towards technology-related products or services [62–66], there has been limited research on exploring the perspectives of users and non-users in a work setting. Most research conducted on the perceptions of users and non-users of new technologies focuses on commercial spheres where people have an individual choice in adopting technologies. Examples of this are studies on the use of GPS by older adults [67], health and fitness applications [68], or mobile banking [69, 70]. Many of these studies use the Technology Acceptance Model theory. Studies using TAM mainly focus on individual users’ attitudes towards and beliefs about using the latest technology-based products or services in their daily lives [68]. The opinions of experienced users or non-users of advanced manufacturing technologies where acceptance is not an individual choice but enforced upon the workforce are often overlooked.

The impact of prior experience with technology on workforce attitudes is complex, shaped by individual differences, and sometimes contradictory. While both formal training and hands-on learning are generally expected to enhance competence, research has shown that this relationship is not always reliable [71]. In terms of technology acceptance, social influence plays a particularly significant role when technology use is mandatory, such as in workplace settings. This influence may be stronger for females and older individuals, although its effect may diminish as users gain more experience and familiarity with the system [72].

Acceptance is also heavily influenced by the nature of users' prior experiences. While increased familiarity with technology is intuitively linked to greater comfort and confidence, this is not always the case if the prior experience has been negative. For example, our own extensive research on electronic voting reveals that individuals with higher technical expertise or greater awareness of system vulnerabilities often develop more critical perspectives, highlighting risks such as software errors, vote accuracy, verification, voter anonymity, and potential for large-scale fraud [73–75]. Similarly, in manufacturing, heightened awareness of operational risks may increase employees’ safety concerns and contribute to hesitancy toward new technologies [11].

Consequently, prior experience does not straightforwardly predict positive acceptance. Instead, it must be understood in context, considering both the content and interpretation of those experiences. Our study explicitly investigates how these individual differences (age, gender, education, and prior experience) shape perceptions of DMTs (RQ2 and RQ4). By comparing technology users and non-users within manufacturing, we provide empirical evidence on how personal factors influence attitudes, acceptance, and perceived barriers. This contributes to developing more nuanced and targeted strategies for technology implementation that are sensitive to the diverse needs and experiences of the workforce.

**Fig. 1:**
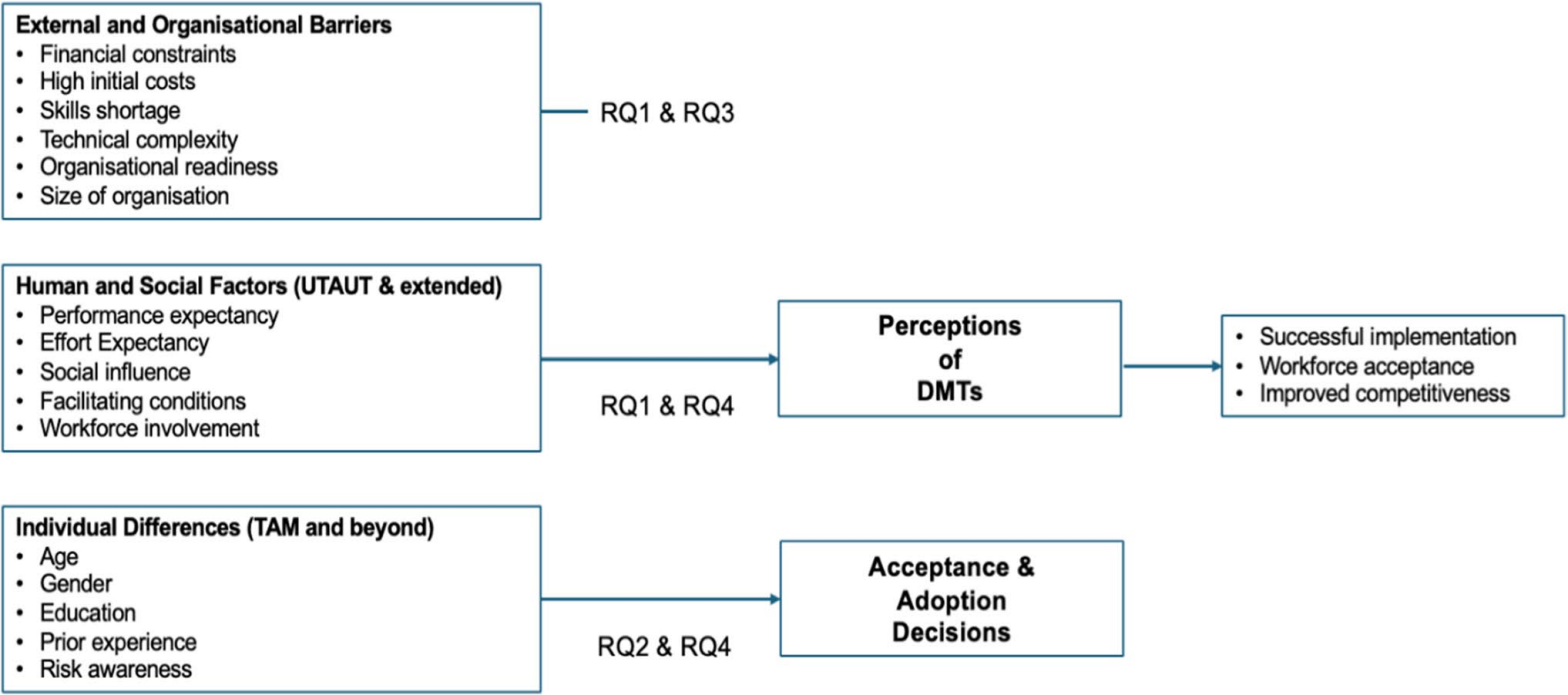
Conceptual framework illustrating the factors influencing workforce perceptions, acceptance, and adoption of digital manufacturing technologies (DMTs) in UK manufacturing

In summary, the theoretical framework presented integrates organisational, social, and individual perspectives to holistically understand the adoption of digital manufacturing technologies. By combining economic and technical barriers with human and individual factors through the lenses of TAM and UTAUT, the framework underscores the complex interplay shaping workforce perceptions and acceptance. This comprehensive approach directly supports the study’s aim to identify the factors influencing employee attitudes toward DMTs and offers a foundation for more human-centred and inclusive adoption strategies. In doing so, it not only bridges existing theoretical gaps but also provides actionable insights for policymakers, managers, and system designers striving to achieve successful digital transformation in the UK manufacturing sector. To visually synthesise the theoretical constructs discussed above, Fig. 1 presents a conceptual framework that integrates organisational, human, and individual factors influencing the acceptance and adoption of digital manufacturing technologies (DMTs). This framework illustrates the relationships between external barriers, human and social determinants, individual differences, and their collective impact on workforce perceptions and adoption decisions. It also clearly shows how these dimensions connect to the research questions guiding this study.

## Research methodology

### Online survey

As part of the EPSRC funded DigiTOP project^1^ and the European H2020 funded AI-PRISM project^2^ we developed a survey study to gather the perceptions and experiences of people working in manufacturing to inform us on the acceptance and adoption of digital manufacturing technologies in British manufacturing and the human factors that may play a part. We addressed different stakeholders working within UK manufacturing companies such as operators, production managers, production technicians, engineers, maintenance technicians, quality inspectors, Human Resources personnel, middle management, senior management and CEOs.

### Recruitment

A total of 313 manufacturing employees participated in the survey, of which 63% were male (further details about the sample provided in “3.3 Participants profile”). The initial survey was reviewed by three experts with experience in manufacturing and survey research. It was then distributed via email to industry contacts and shared in relevant LinkedIn groups and on Facebook, yielding 13 responses over 38 days. These responses were analysed and discussed with the reviewers as part of a pilot study, which assessed the survey’s logic, relevance, and content validity. No changes were deemed necessary, and final data collection proceeded as planned. To increase the response rate and be guaranteed of sufficient respondents, we used the recruitment platform Prolific Academic.^3^ Prolific is a platform for online subject recruitment established in 2014, which explicitly caters to researchers and provides a pool of 70,000 + vetted participants. A study comparing Prolific to two other platforms for crowdsourcing online human-subjects research (i.e. Amazon Mechanical Turk and CrowdFlower) found that Prolific participants reported higher naivety than MTurk participants [76], meaning that they have a lower level of familiarity with commonly used research materials because their prior exposure to research is limited [77] and are therefore less likely to potentially bias the results of the studies. Furthermore, Prolific’s participants provide high data quality, show lower degrees of dishonest behaviour, and were also more diverse in terms of geographical location and ethnicity than MTurk participants [76, 78]. The platform offers the opportunity to filter certain demographics to pre-screen the participants. In our case we needed UK participants working in manufacturing. The platform allowed us to easily integrate our Qualtrics survey tool. It took 15 h to gather the data from 300 qualifying respondents. The final study sample consisted of 13 pilot participants and 300 Prolific participants.

Participants completed the survey online, which was timed to take no longer than 12 min. The survey was organised in three parts. The first section investigated the perceptions of all manufacturing employees on digital manufacturing technologies (*N* = 313). In the second part, employees who worked in organisations that use DMTs such as robots, virtual reality or sensors were questioned about their experience with these technologies and other related issues (*N* = 184). In the final section all participants provided demographic information. This paper focuses primarily on the perceptions of the 313 employees.

Participants took an average of 527 s (just under 9 min) to complete the study (SD = 321 s), with an average payment equivalent to £7.50 per hour. The study was approved by Cranfield University’s Research Ethics System, and all participants provided informed consent before participating.

### Participants profile

**Fig. 2:**
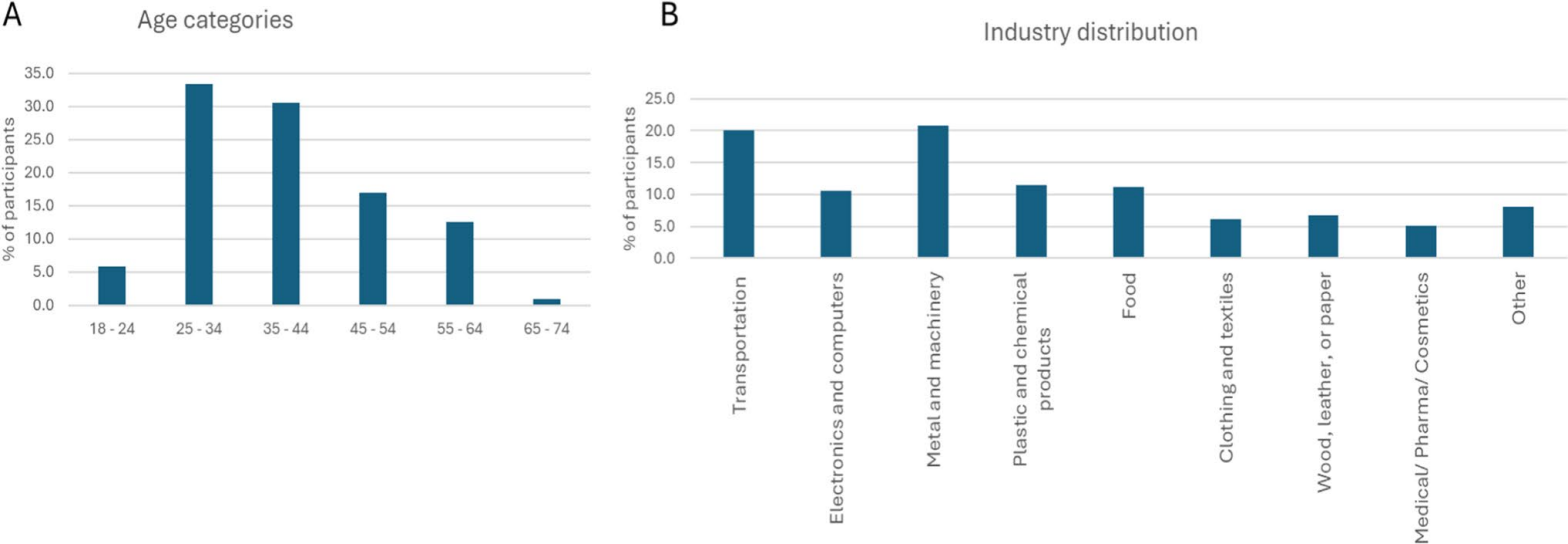
**A** Participants age distribution; **B** Participant distribution according to industry they work in

Of the 313 manufacturing employees surveyed, 196 were male, 115 were female, 1 selected “prefer not to say,” and 1 did not respond to the gender question. Most participants fell within the age groups of 25–34 (33.3%) and 35–44 (30.4%). (Fig. 2 A). A total of 184 participants reported using digital manufacturing technologies (DMTs), while 129 indicated that their companies did not utilise DMTs in their work processes. Participants represented various manufacturing industries: 20.8% worked in metal and machinery, 20.1% in transportation, 11.5% in plastics and chemicals, 11.2% in food and beverages, 10.5% in electrical/electronics, 6.7% in wood, leather, or paper, 6.1% in clothing and textiles, 5.1% in medical/pharma/cosmetics, and 8% selected "Other" (Fig. 2 B).

Regarding roles, participants initially chose from 13 options, including operator, production manager, technician, engineer, quality inspector, HR/admin/finance, maintenance technician, middle or senior management, CEO, customer service, marketing/sales, and other. These were consolidated into three main categories: 53.5% shop floor workers (e.g. operators, technicians, engineers, inspectors), 45.7% managerial roles (e.g. middle and senior management, CEOs), and 0.8% customer-facing roles (e.g. customer service, marketing/sales). Participants reported an average tenure of 7.68 years (SD = 6.67) in their current positions.

The sample also showed a balanced distribution of company sizes: 31% worked in small companies (fewer than 100 employees), 30% in medium-sized companies (100–500 employees), and 39% in large companies (over 500 employees).

In terms of education, 50.8% held a college or university degree, 20.6% had higher or further education, 17.7% possessed a post-doctoral degree, and 10.6% had completed secondary school up to 16 years of age.

### Analysis

The collected data was exported to SPSS Statistics 26. The data was checked for incomplete responses and completion times shorter than 3 min. The first step for the data analysis was an overview of the participants’ responses as a whole group. A non-parametric Wilcoxon Signed Rank test with hypothetical median of 3 (middle score on all answer options) was used to establish whether participants’ responses differed significantly from the neutral answer option. Following this an exploratory factor analysis was used to reduce the number of potential variables. All 31 items of five questions of interest were included in this analysis. We then assessed the reliability of all resulting factors using Cronbach’s alpha. Alpha above 0.7 was considered reliable and accepted for further analysis. The second step of the analysis was to compare the perceptions of DMTs depending on participants’ characteristics (gender, education, age, role in the company, company size, usage of DMTs). As the data was ordinal and not normally distributed (Kolmogorov–Smirnov < 0. 35 and Shapiro Wilk statistics < 0.94, *p* < 0.001 for both statistics), non-parametric test were applied. A Kruskall Wallis test for independent samples was used to assess whether there was a significant difference between three or more participant groups, and a further post hoc analysis with Mann–Whitney was used to investigate where these differences were observed. The multiple comparisons were adjusted with a Bonferroni correction as specified with each analysis. For the analysis with two participant groups (gender, DMT users versus non-users) a non-parametric Mann–Whitney test for two independent samples was applied.

## Results

### Employee perceptions on digital manufacturing technologies

To start answering RQ1, a general overview of the sample was produced to understand the views of manufacturing employees towards DMTs. Across the sample, most participants agreed or strongly agreed that using digital manufacturing technologies (DMTs) would enhance a company’s productivity (86.9%) and improve product quality (80.7%). Additionally, DMTs were widely seen as beneficial for maintaining competitiveness (84%) and reducing costs (71.8%).

However, participants were less confident about physical interaction with new technologies. Agreement that staff could easily interact with newly adopted DMTs or learn to operate them was lower, at 50.8% and 54.8%, respectively. Moreover, 19.5% and 19.2% of participants disagreed with these statements.

**Fig. 3:**
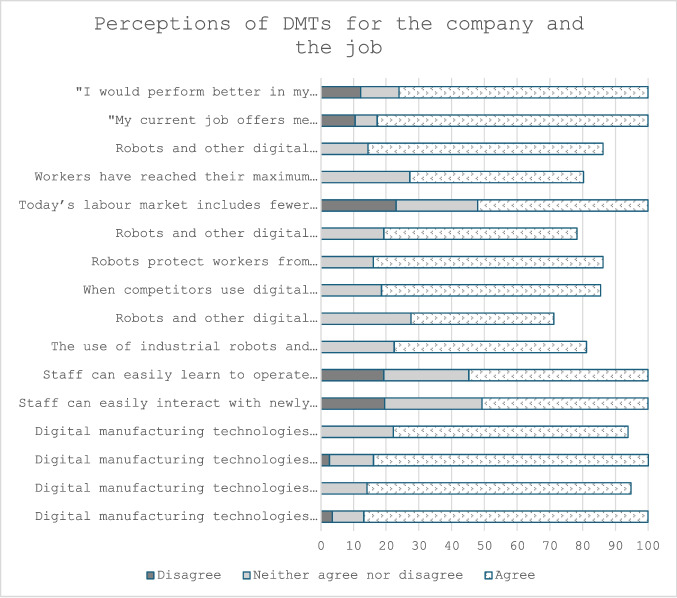
Agreement/disagreement percentage for items relating to perceptions on DMTs for the company and the job

Regarding job roles, a large majority of participants felt that their current job provided sufficient opportunities to use their knowledge and skills (82.8%). At the same time, 76.1% agreed that gaining additional skills and knowledge would improve their performance (Table 1, Fig. 3).

**Table 1:** Inferential statistics for items relating to perceptions on DMTs for the company and the job

Items	One sample Wilcoxon Signed Rank Test
- Digital manufacturing technologies enhance a company’s productivity	Z = 13.92, *n* = 313, *p* ≤ 0.001
- Digital manufacturing technologies improve product quality	Z = 13.34, *n* = 312, *p* ≤ 0.001
- Digital manufacturing technologies are useful to maintain competitiveness	Z = 14.01, *n* = 312, *p* ≤ 0.001
- Digital manufacturing technologies reduce costs	Z = 12.29, *n* = 312, *p* ≤ 0.001
- Staff can easily interact with newly adopted digital manufacturing technologies	Z = 6.95, *n* = 313, *p* ≤ 0.001
- Staff can easily learn to operate newly adopted digital manufacturing technologies	Z = 7.62, *n* = 312, *p* ≤ 0.001
- The use of industrial robots and other digital manufacturing technologies creates more desirable jobs, such as engineering, programming, equipment maintenance, and management	Z = 8.43, *n* = 313, *p* ≤ 0.001
- Robots and other digital manufacturing technologies help to create UK jobs by re-shoring more manufacturing work	Z = 3.84, *n* = 313, *p* ≤ 0.001
- When competitors use digital manufacturing technologies you have to follow the trend	Z = 10.21, n = 313, p ≤ 0.001
- Robots protect workers from repetitive, mundane, and dangerous tasks	Z = 10.86, *n* = 313, *p* ≤ 0.001
- Robots and other digital manufacturing technologies free up manpower to let companies maximise workers' skills in other areas of the business	Z = 7.43, *n* = 313, *p* ≤ 0.001
- Today’s labour market includes fewer skilled manufacturing workers due to decades of offshoring. Robots eliminate this shortfall	Z = 5.66, *n* = 313, *p* ≤ 0.001
- Workers have reached their maximum output capacity, and digital manufacturing technologies can help manufacturers break that ceiling	Z = 6.12, *n* = 313, *p* ≤ 0.001
- Robots and other digital manufacturing technologies will replace unskilled workers	Z = 10.96, *n* = 313, *p* ≤ 0.001
- "My current job offers me sufficient opportunity to use my knowledge and skills"	Z = 12.59, *n* = 313, *p* ≤ 0.001
- "I would perform better in my current job if I possessed additional knowledge and skills”	Z = 11.40, *n* = 313, *p* ≤ 0.001

Participants were less optimistic about the impact of digital manufacturing technologies (DMTs) on jobs compared to their overall perception of these technologies (Table 1, Fig. 3). While 58.8% agreed that DMTs create more desirable jobs, and 70.3% agreed that robots protect workers from mundane, repetitive, and dangerous tasks, opinions were more divided on issues related to offshoring and workforce demands.

Regarding reshoring, 43.7% agreed that DMTs could create more UK jobs by bringing manufacturing work back, while 27.5% were neutral, and 28.7% disagreed. Similarly, 52.1% agreed that robots could address the shortfall of skilled manufacturing workers, but 24.9% were neutral, and 23% disagreed.

When asked about the effect of advanced robotics, virtual/augmented reality, and other DMTs on job expertise requirements, 53.5% believed the required expertise for their roles would remain the same. Meanwhile, 31.6% expected expertise requirements to increase, and 14.8% thought they would decrease.

In examining concerns associated with digital manufacturing technologies (DMTs), the data indicate that key apprehensions centred around the allocation of substantial resources for maintenance (43.3% reported being extremely or very concerned) and the underestimation of costs and development time associated with new technologies (41.3% extremely or very concerned).

**Fig. 4:**
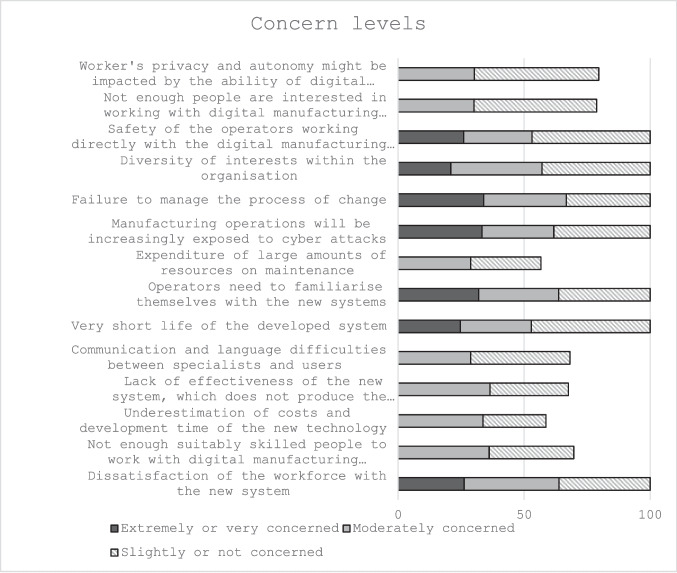
Participants concern level (%) with regards to the effect of DMTs on the company and the workforce

Conversely, participants expressed lower levels of concern regarding several other issues. Specifically, a significant proportion reported being only slightly or not at all concerned about a lack of interest in working with DMTs (48.7%), workers’ privacy and autonomy (49.3%), the potentially short lifespan of developed systems (47.1%), operator safety when directly interacting with these technologies (46.8%), and the diversity of interests within organisations (42.9%). A detailed summary of these perceptions is presented in Table 2 and Fig. 4.

**Table 2:** Inferential statistics for participants concern level with regards to the effect of DMTs on the company and the workforce

	One sample Wilcoxon Signed Rank Test
- Dissatisfaction of the workforce with the new system	Z = 2.46, *n* = 313, *p* ≤ 0.014
- Not enough suitably skilled people to work with digital manufacturing technologies	Z = 0.93, *n* = 313, *p* = 0.354
- Underestimation of costs and development time of the new technology	Z = −2.44, *n* = 312, *p* = 0.015
- Lack of effectiveness of the new system, which does not produce the expected results	Z = 0.39, *n* = 312, *p* = 0.694
- Communication and language difficulties between specialists and users	Z = 2.72, *n* = 312, *p* = 0.007
- Very short life of the developed system	Z = 5.57, *n* = 312, *p* ≤ 0.001
- Operators need to familiarise themselves with the new systems	Z = 0.59, *n* = 312, *p* = 0.554
- Expenditure of large amounts of resources on maintenance	Z = −3.09, n = 312, *p* = 0.002
- Manufacturing operations will be increasingly exposed to cyber attacks	Z = 1.14, *n* = 312, p = 0.255
- Failure to manage the process of change	Z = 0.22, *n* = 312, *p* = 0.825
- Diversity of interests within the organisation	Z = 5.12, *n* = 312, *p* ≤ 0.001
- Safety of the operators working directly with the digital manufacturing technologies such as robots	Z = 4.56, *n* = 312, *p* ≤ 0.001
- Not enough people are interested in working with digital manufacturing technologies	Z = 6.99, *n* = 312, *p* ≤ 0.001
- Worker's privacy and autonomy might be impacted by the ability of digital manufacturing technologies to collect and monitor real-time performance data from equipment, human operators, and products	Z = 6.11, *n* = 300, *p* ≤ 0.001

As a further step factor analysis was used to reduce the number of possible variables for the analysis.

### Factor analysis

Thirty-one questions relating to perceptions on DMTs were factor analysed using principal components analysis with Oblimin rotation. The Kaiser–Meyer–Olkin measure of sampling adequacy was 0.81, and Bartlett’s test of sphericity was significant (χ2 (496) = 2720.16, *p* < 0.001). Using both the scree plot and eigenvalues > 1 to determine the underlying components, the analysis yielded four factors explaining a total of 44% of the variance in the data. After the analysis of factor loadings, Factor 1 was labelled as “Organisational Barriers”, Factor 2 as “Perceived Usefulness”, Factor 3 as “Effects on employment and work life”, and Factor 4 was labelled as “Perceived Ease of Use” (Table 3). The items in Factors 2 and 4 were adapted from the Technology Acceptance Model.

**Table 3:** Four factor loadings with percentage variance explained and reliability scores (Cronbach’s alpha)

	Factor			
	1	2	3	4
Lack of effectiveness of the new system, which does not produce the expected results	0.765			
Very short life of the developed system	0.697			
Communication and language difficulties between specialists and users	0.695			
Expenditure of large amounts of resources on maintenance	0.665			
Underestimation of costs and development time of the new technology	0.633			
Operators need to familiarise themselves with the new systems	0.557			
Digital manufacturing technologies are useful to maintain competitiveness		0.843		
Digital manufacturing technologies improve product quality		0.769		
Digital manufacturing technologies enhance a company’s productivity		0.765		
Digital manufacturing technologies reduce costs		0.641		
Robots and other digital manufacturing technologies help to create UK jobs by re-shoring more manufacturing work			−0.838	
The use of industrial robots and other digital manufacturing technologies creates more desirable jobs, such as engineering, programming, equipment maintenance, and management			−0.752	
Robots and other digital manufacturing technologies free up manpower to let companies maximise workers' skills in other areas of the business			−0.737	
Robots protect workers from repetitive, mundane, and dangerous tasks			−0.677	
Staff can easily learn to operate newly adopted digital manufacturing technologies				0.841
Staff can easily interact with newly adopted digital manufacturing technologies				0.831
Total variance explained	17.98%	14.64%	5.86%	5.34%
Cronbach's alpha	0.793	0.796	0.816	0.85

To explore how these DMT perceptions differ between participants, an independent sample non-parametric analysis was performed comparing perceptions between different age groups, education levels, gender, role in the company, and whether participants worked in a company that uses digital manufacturing technologies or not.

### Perception differences related to characteristics

To answer the RQ2, the data was analysed by splitting participants into subcategories depending on their characteristics: age, gender, education, organisational role. The size of the company they work for was used for a follow on analysis to answer RQ3.

First, our analysis shows that participant perceptions do not significantly differ depending on age (Kruskal Wallis H(4) ≤ 8.17, *p* ≥ 0.085).

Second, the comparison between male and female participants with the non-parametric two sample Mann Whitney test showed that males scored significantly more positive on Factor 1 “Organisational Barriers” (U = 9576.00, *p* = 0.026) and were more concerned about the dissatisfaction of the workforce with the new system (U = 9480.50, *p* = 0.033). However, the responses to any of the other questions were not statistically significant (U ≥ 10063.00, *p* ≥ 0.104).

Third, comparing different education levels showed that participants’ opinions differ on Factor 4 (“Perceived Ease of Use”: H(3) = 15.77, *p* = 0.001, and Factor 2 (“Perceived Usefulness”: H(3) = 9.05, *p* = 0.029. Other comparisons did not yield a statistically significant result (H(5) ≤ 4.44, *p* ≥ 0.217). To explore where these significant differences were observed, a Mann Whitney test for two independent groups was used with a Bonferroni multiple comparison correction (two-tailed alpha = 0.008). The Factor 4 “Perceived Ease of Use” analysis showed that participants with a secondary education level had significantly lower scores compared to further education, college or university and postgraduate degree education participants (U = 718.50, *p* = 0.006, U = 1694.00, *p* = 0.001, and U = 472.50, *p* ≤ 0.001, respectively), meaning that secondary school education participants disagreed more on the statements assessing ease of use of DMTs. The other differences between education levels were not significant (U ≥ 1513.50, *p* ≥ 0.186). Equivalent analysis on the Factor 2 “Perceived Usefulness” showed that secondary school had significantly lower scores only compared to college or university education participants: college and university education level participants perceived DMTs as significantly more useful (U = 1894.00, *p* = 0.007). The other differences were not significant after the Bonferroni correction (U ≥ 683.00, *p* ≥ 0.012).

Fourth, an equivalent analysis on participant perceptions depending on their organisational role (shop floor vs. managerial vs. customer facing) showed that there were no significant differences (H(2) ≤ 3.35, *p* ≥ 0.188. Yet, looking at more detailed roles, and in particular the ones directly related to the DMT technology (operator, production technician, production manager, engineer, maintenance technician, middle management, senior management and CEO) and correlating these positions, with the derived factors and questions asking their agreement with statements "My current job offers me sufficient opportunity to use my knowledge and skills", "I would perform better in my current job if I possessed additional knowledge and skills” and “Would introduction of advanced robotics, virtual/augmented reality, or other digital manufacturing technologies increase or decrease the required expertise for your job?”. The results showed a significant negative correlation with Factor 1 “Organisational Barriers” (Spearman rho = −0.153, *p* = 0.026), suggesting that a higher position was related to lower scores on this factor. There was a significant positive correlation between job position and the statement that the current job provides opportunities to use knowledge and skills (rho = 0.160, *p* = 0.020).

Finally, analysis on the company size (small vs. medium vs. large) showed only a significant difference in Factor 4 “Perceived Ease of Use” (H(2) = 6.58, *p* = 0.037) and on Q13 whether the introduction of advanced robotics, virtual/augmented reality or other digital manufacturing technologies would increase or decrease the required expertise for the job (H(2) = 8.33, *p* = 0.016). The post hoc analysis with a Bonferroni correction (2-tailed alpha = 0.016) showed that in Factor 2 “Perceived Usefulness” employees of large companies have significantly more positive perceptions on Perceived Ease of Use compared to employees of small companies (U = 4748.50, *p* = 0.012), though the two other comparisons (large vs. medium and medium vs. small) did not differ significantly (U ≥ 4045.00, *p* ≥ 0.176). Regarding Q13, employees from large companies were significantly more likely to believe that the introduction of DMTs would increase the expertise required to perform their jobs compared to employees from medium-sized and small companies (U = 4325.00, *p* = 0.013 and U = 4326.00, *p* = 0.015, respectively), however there was no statistically significant difference between medium and small companies (U = 3998.00, *p* = 0.869).

Although we observed some small differences in perceptions between gender, education levels, company role, and company size, the main differences were observed when we compared the perceptions of the DMT user and non-user groups. The next section will explore these differences more in depth.

### Perception differences between DMT users and non-users

While investigating RQ4, our results showed that there are significant statistical differences in demographic characteristics and perceptions between those who work in manufacturing companies that use digital manufacturing technology and those that don’t. The participants who work in industries where no DMTs have been adopted will be referred to as ‘DMT non-users’ (*N* = 129). For brevity, we will refer to the participants who work in a company where any DMT has been implemented as ‘DMT users’ (*N* = 184) even though this does not necessarily imply that they have hands-on experience in using these technologies. However, they may have been involved in the acquisition of the technology, in organising the training for staff, or in dealing with the vendors. Or they have seen the technology been used by colleagues and have therefore some familiarity with it.

Further analysis explored the differences between these two participant groups, first comparing the users and non-users to their demographic information. The non-parametric independent samples Mann–Whitney U test showed that users and non-users did not significantly differ in age, their role in the company, or years working in the company (U > 10,975.00, *p* > 0.256). However, there were significant differences in gender distribution: there were more males in the DMT user group while DMT non-user group had a similar number of males and females (U = 9586.50, *p* = 0.001); DMT users reported significantly higher levels of education compared to the DMT non-users (U = 9285.50, *p* = 0.001) and there were significantly more DMT users in large companies, while the DMT non-users worked mainly in small sized companies (U = 7358.50, *p* < 0.001, Table 4).

**Table 4:** Demographic information as a function of DMT use or non-use

		DMT users		DMT non-users	
Background variable	Category	Frequency	(%)	Frequency	(%)
Gender*	Male	129	41	67	22
	Female	54	17	61	20
	Prefer not to say	0	0	1	0.3
Age group	18—24	12	4	6	2
	25—34	57	18	47	15
	35—44	57	18	38	12
	45—54	34	11	19	6
	55—64	23	7	16	15
	65—74	0	0	3	1
Education*	Secondary up to 16 years	5	2	29	9
	Higher/further education	39	13	25	8
	College or university	105	34	53	17
	Post-graduate degree	34	11	21	7
Role in the company	Shop floor	82	32	56	22
	Managerial	73	28	45	17
	Customer facing	1	0.4	1	0.4
Company size* Industry	Small (< 100 employees)	35	11	62	20
	Medium (100–500 employees)	55	18	39	13
	Large (> 500 employees)	94	30	28	9
	Transportation	50	16	13	4
	Electrical/Electronics	20	6	13	4
	Metal and machinery	31	10	34	11
	Plastic and chemical products	18	6	18	6
	Food and beverages	22	7	13	4
	Clothing and textiles	10	3	9	3
	Wood, leather, or paper	12	4	9	3
	Medical/Pharma/Cosmetics	10	3	6	2
	Other	11	4	14	5
		mean	std	mean	std
Years in the company		8.07	6.85	7.12	6.38

The next step of the analysis concerned the differences in DMT perception between the two groups of interest. Non-parametric Mann–Whitney U test showed that users had significantly more positive attitudes on Perceived Usefulness, Effects on employment and work life, and Perceive Ease of Use of DMTs (U = 8792.00, *p* < 0.001, U = 8258.00, *p* < 0.001, and U = 10268.500, *p* = 0.038, respectively). Opinions on the question whether the introduction of advanced robotics, virtual/augmented reality, or other digital manufacturing technologies would increase or decrease the required expertise for the job also differed significantly between the two groups of participants (U = 8548.00, *p* = 0.002). The majority (53.3%) of the DMT non-users indicated that the required expertise would not be affected by this technology, while 24.2% indicated an increase and 22.5% a decrease. In contrast, the DMT users, although also predominantly indicating that there will be no change (53.7%), were more often than the non-users of the opinion that the introduction of DMTs will result in an increase in required expertise (36.7%). Furthermore, the DMT users were more often in agreement with the statement that “Today’s labour market includes fewer skilled manufacturing workers due to decades of offshoring. Robots eliminate this shortfall” (U = 10,257.00, *p* = 0.039, Table 5).

**Table 5:** The mean scores (std) for perceptions of DMT by user and non-user groups

	DMT users	DMT non-users
F1: Organisational Barriers	3.03 (0.77)	3.00 (0.78)
**F2: Perceived Usefulness	3.66 (0.82)	3.32 (0.77)
**F3: Effects on employment and work life	4.16 (0.65)	3.88 (0.60)
*F4: Perceived Ease of Use	3.51 (0.89)	3.31 (0.85)
*Today’s labour market includes fewer skilled manufacturing workers due to decades of offshoring. Robots eliminate this shortfall	3.48 (1.01)	3.20 (1.13)
Workers have reached their maximum output capacity, and digital manufacturing technologies can help manufacturers break that ceiling	3.45 (1.04)	3.33 (1.07)
Robots and other digital manufacturing technologies will replace unskilled workers	3.83 (1.06)	3.87 (1.00)
"My current job offers me sufficient opportunity to use my knowledge and skills"	4.10 (0.99)	4.03 (0.98)
"I would perform better in my current job if I possessed additional knowledge and skills”	3.99 (0.98)	3.85 (1.13)
**Would the introduction of advanced robotics, virtual/augmented reality, or other digital manufacturing technologies increase or decrease the required expertise for your job? (1 = increase, 2 = stay the same, 3 = decrease)	1.73 (0.63)	1.98 (0.69)

These are the average responses from a scale of 1 (strongly disagree) to 5 (strongly agree), **/* denotes that the figure is significantly different to the rest of the sample at the **p < 0.001, *p < 0.05 respectively

When we look at the concerns participants might have in relation to the new technologies, we see a trend significant difference related to the safety of operators working directly with the digital manufacturing technologies such as robots, with DMT users being more concerned (U = 10473.00, *p* = 0.095). Other comparisons did not yield significant differences between two participant groups (U > 10632.00, *p* > 0.0144, Table 6).

**Table 6:** The mean scores (std) for concern related to DMTs by users and non-users

	DMT users	DMT non-users
Dissatisfaction of the workforce with the new system	3.17 (0.99)	3.11 (1.15)
Not enough suitably skilled people to work with digital manufacturing technologies	3.08 (1.06)	3.02 (1.09)
Manufacturing operations will be increasingly exposed to cyber attacks	3.05 (1.21)	3.12 (1.20)
Diversity of interests within the organisation	3.26 (1.03)	3.4 (1.06)
˟Safety of the operators working directly with the digital manufacturing technologies such as robots	3.22 (1.19)	3.45 (1.13)
Not enough people are interested in working with digital manufacturing technologies	3.56 (1.13)	3.37 (1.19)
Worker's privacy and autonomy might be impacted by the ability of digital manufacturing technologies to collect and monitor real-time performance data from equipment, human operators, and products	3.45 (1.12)	3.43 (1.19)

These are the average responses to a scale of 1 (Extremely concerned) to 5 (Not concerned at all). ˟ denotes ˟*p* < 0.1 levels of significance

## Discussion

The principal aim of this study was to explore manufacturing workers’ perceptions of factors influencing the acceptance and adoption of digital manufacturing technologies (DMTs), addressing four specific research questions. Our findings shed light not only on the broad alignment between employee perceptions and the academic literature but also reveal nuanced insights that challenge or deepen existing assumptions.

First, regarding whether manufacturing employees identify the same advantages and barriers to DMT implementation as described in the literature (RQ1), our results indicate overall support for established benefits, such as productivity improvements, better product quality, and strengthened competitiveness. They also view these technologies as easy to use and believe they will positively impact employment by creating more skilled and desirable jobs, while reducing repetitive, mundane, and hazardous tasks. Yet, employees expressed significant concerns about economic factors, especially ongoing maintenance costs and underestimated development time. Interestingly, concerns over safety, privacy, and autonomy were minimal, which suggests a possible disconnect between scholarly emphasis on ethical and human-centric design principles (as emphasised in Industry 5.0 discourse) and the practical priorities of workers on the shop floor. This might reflect that immediate economic and operational concerns are more tangible to employees than abstract ethical issues, highlighting a potential gap that both researchers and managers should address when designing communication and training strategies.

In examining whether perceptions are linked to individual characteristics (RQ2), we found few differences by gender and age, supporting recent research that challenges stereotypes of older workers as more resistant to technological change [56]. However, educational background and hierarchical position significantly shaped perceptions. We found that the higher the position in the company (management, CEOs) the more concern was raised about issues such as underestimation of cost, communication difficulties between specialists and users, and lack of effectiveness (i.e. the technology does not produce the expected results). Lower-educated workers were more sceptical about DMTs’ impact on employment and work quality, likely reflecting a heightened sense of vulnerability [27]. This suggests that while technological optimism might prevail in strategic discourse, frontline anxieties persist and must be addressed through targeted reskilling efforts and transparent communication to mitigate fears of displacement.

Regarding the size of the organisation (RQ3), our data confirmed that employees in large firms expressed more positive views of DMTs and were more likely to believe these technologies create desirable jobs. While this finding might reflect larger firms’ greater financial capacity and resource availability [79], it also underscores the scalability gap that SMEs face, where economic constraints and limited support structures hinder both adoption and workforce confidence. Policymakers and industry leaders must therefore consider tailored interventions for SMEs, recognising that enthusiasm for technological change is deeply intertwined with organisational capacity.

When comparing DMT users and non-users (RQ4), we observed more positive attitudes among users, consistent with experiential learning theories suggesting that direct engagement fosters more favourable perceptions. Actual experience with the new technologies has shaped their view and we found that those working in companies with digital manufacturing technologies indicate that their skills, autonomy, and the number of interesting tasks has increased, while their levels of fatigue, physical workload, and stress decreased. However, a critical nuance emerges: DMT users were also more concerned about safety, likely due to firsthand exposure to technical failures and unexpected operational challenges [11]. This duality, heightened optimism about benefits alongside greater awareness of risks, suggests that simplistic narratives framing technology users as uniformly enthusiastic are misleading. It underscores the importance of involving experienced operators in feedback loops and safety planning, rather than assuming technical familiarity guarantees uncritical acceptance.

A particularly striking finding is that many operators and non-users did not perceive a need for upskilling in response to DMT implementation, contradicting widespread scholarly and policy claims that future manufacturing work will demand higher-level cognitive and technical skills. This mismatch points to a critical misalignment between workforce self-perception and actual skill demands forecasted by research and industry roadmaps. The OECD [80] for instance, finds mismatches both in the skills of individuals and in the educational credentials they hold, compared with what companies need. While basic cognitive, physical and manual skills will be in lesser demand, there will be an increased demand for higher cognitive skills (e.g. creativity, critical thinking, decision making, and complex information processing), social and emotional skills, and technological skills [81]. Such a gap raises concerns about future readiness and highlights the urgent need for proactive, accessible reskilling programs, particularly for lower-skilled or less formally educated workers who may underestimate future skill requirements. Furthermore, the high proportion of respondents (83%) reporting sufficient opportunities to use their skills contrasts with broader evidence of skill mismatches reported in global studies. This suggests that while some employees feel adequately engaged in their current roles, there may be an overestimation of skill utilisation or a lack of awareness regarding latent skill gaps, particularly in rapidly evolving technological contexts. This complacency could impede proactive learning and adaptation, risking both individual and organisational competitiveness.

Taken together, these findings not only validate known enablers and barriers but also emphasise the complex, often contradictory ways in which employees interpret technological change. Rather than being passive recipients of top-down innovation strategies, workers actively negotiate their sense of security, value, and future relevance. A more critical reflection reveals that successful DMT adoption cannot rely solely on economic incentives or technological readiness. It requires ongoing dialogue with employees, nuanced understanding of psychological and social dynamics, and concrete support mechanisms that align perceived and actual skill demands. Our study thus contributes to advancing theoretical and practical understanding by foregrounding the human dimension of digital transformation in manufacturing. It highlights the importance of addressing perceptual and experiential differences within the workforce, moving beyond generalised assumptions about technological optimism or resistance. By integrating these nuanced perspectives, future interventions can be more equitable, inclusive, and ultimately more effective.

## Conclusions

The primary aim of this survey study was to investigate manufacturing workers’ perceptions of factors influencing the acceptance and adoption of digital manufacturing technologies (DMTs), while comparing these perceptions between users and non-users and exploring how individual and organisational characteristics shape these views. Our findings reveal that manufacturing employees generally hold positive perceptions of DMTs, particularly among those with direct experience using them. These results underscore the importance of aligning technological advancements with human involvement and empowerment to facilitate seamless integration of DMTs in manufacturing environments.

Theoretically, this study advances current understanding in several important ways. First, by integrating insights from the Technology Acceptance Model (TAM) and the Unified Theory of Acceptance and Use of Technology (UTAUT), and explicitly including individual factors such as education level, job position, and prior experience, our research addresses a significant gap in technology adoption literature: the underexplored role of human and organisational context in shaping technology acceptance within industrial, non-voluntary adoption settings. Whereas existing models often focus on voluntary consumer adoption or managerial perspectives, our study uniquely centres on frontline manufacturing workers, providing empirical evidence of how perceptions evolve through practical exposure and organisational context.

Furthermore, our study highlights the critical role of experiential learning and familiarity in shaping positive attitudes toward DMTs. These key aspects are often underemphasised in traditional acceptance frameworks. By demonstrating that workers with direct experience report higher perceived usefulness and ease of use, we extend theoretical models by emphasising the importance of "trialability" and participatory involvement as moderating factors in technology acceptance [82]. These insights suggest that experiential dimensions should be more formally integrated into future iterations of technology adoption theories, particularly for workplace settings where adoption is often top-down and mandatory.

Additionally, our findings contribute to the emerging theoretical discourse surrounding Industry 5.0 by reinforcing the need for a human-centric perspective that moves beyond efficiency to prioritise well-being, empowerment, and skill development. Our results support and deepen the conceptual foundations of human-centric innovation, illustrating how inclusive design and early involvement of shop floor employees can reduce resistance and facilitate smoother transitions. This aligns with and extends the “design for all” principle, providing empirical evidence that can inform both theoretical refinement and practical strategies.

In conclusion, this research contributes to bridging the gap between technological potential and human factors in manufacturing. By providing a nuanced understanding of employee perceptions and identifying the mechanisms through which these perceptions are shaped, our study offers a robust foundation for both future theoretical development and practical interventions. Moving forward, deeper investigations into how different sub-groups experience and negotiate technological change will be critical to developing truly inclusive and effective digital transformation strategies in line with Industry 5.0 ideals.

## Limitations and future research

Despite its contributions, this study has several limitations that should be acknowledged and can inform future research directions. First, although we compared perceptions between DMT users and non-users, we did not capture the depth or nature of participants’ exposure to these technologies. It remains unclear whether the “users” in our sample had frequent, hands-on operational experience or merely observed DMTs in use within their work environment. This distinction is crucial, as direct interaction may shape perceptions and attitudes differently from indirect exposure. Future studies should explore these nuances more thoroughly, for example by including more detailed survey questions or incorporating qualitative methods to better capture varying levels of engagement.

Second, the study grouped all DMTs under a single umbrella, without distinguishing between specific types (e.g. robotics, additive manufacturing, augmented reality). Different technologies might elicit varying perceptions regarding usefulness, ease of use, safety, and job impact. Disaggregating DMT types in future research could uncover technology-specific concerns and advantages, allowing for more tailored implementation strategies.

Third, the observed positive correlation between DMT adoption and higher education levels raises important questions about causality and workforce dynamics. It remains unclear whether higher education predisposes individuals to adopt and embrace advanced technologies or whether working in technologically advanced environments incentivises further education and skill development. Longitudinal or mixed-methods studies could help disentangle these relationships and shed light on how DMT exposure might influence educational aspirations and career trajectories, particularly among younger workers. Furthermore, incorporating additional demographic variables could provide a more comprehensive understanding of individual characteristics and attitudes toward DMT. Family status, although traditionally studied in relation to the use of digital technologies for education and children/adolescent development may also influence an individual’s exposure to technology and shape their attitudes toward DMT [83, 84]. While the literature directly linking family status to DMT remains limited, there is evidence suggesting that socio-economic status (SES), which is often correlated with education levels and is discussed above, plays a role; for instance, workers with higher SES tend to have greater access to and more use of information and communication technologies (ICT) [85]. Existing studies have primarily focused on how company policies, particularly in family-owned businesses, reflect the leadership’s attitudes toward technology and their willingness to adopt novel technologies [86]. However, the extent to which these organisational policies influence the attitudes of individual operators remains underexplored and should be addressed in future research.

Moreover, one unexpected and particularly intriguing finding was that non-users did not anticipate a need for upskilling, despite simultaneously agreeing that DMTs would likely replace unskilled labour. This contradictory view suggests possible gaps in understanding future skill requirements and underlines a disconnect between perceived and actual demands of digital transformation. Qualitative approaches, such as in-depth interviews, ethnographic studies, or focus groups, could help unpack these inconsistencies by exploring underlying fears, misconceptions, and cultural narratives that shape these beliefs.

Additionally, while this study focused primarily on perceptions of benefits and barriers, it did not deeply examine the emotional responses or sources of enthusiasm and anxiety experienced by current DMT users. Future research should investigate the psychological and emotional dimensions of DMT adoption, including factors such as trust in technology, job satisfaction, perceived autonomy, and feelings of job security. Understanding these dimensions would offer a more holistic view of adoption readiness and workforce well-being.

Furthermore, we are planning a large-scale follow-up study specifically focusing on robot use in manufacturing. This future research will allow us to examine in greater detail how different levels of direct interaction with robotics, as well as specific application contexts, shape employees’ perceptions, attitudes, and readiness for adoption. By focusing on robotics as a distinct and rapidly growing subset of DMTs, we aim to generate more targeted insights that can inform both theory and practice.

Lastly, there is a need for cross-cultural and cross-sectoral studies to determine whether these findings generalise beyond the UK manufacturing context. Comparative research across countries or different industrial sectors could illuminate cultural, regulatory, and market-specific factors that shape technology acceptance.

In summary, while our study underscores the importance of experience, engagement, and participation in shaping perceptions of DMTs, it also highlights several critical areas requiring further exploration. Addressing these gaps can enable more targeted, inclusive, and effective strategies for human-centred technological transformation. Empowering employees through transparent communication, early involvement, and ongoing support will be essential to realising the full transformative potential of digital manufacturing technologies, particularly within the evolving framework of Industry 5.0.

## Abbreviations

DMT: Digital Manufacturing Technologies
SME: Small and Medium-sized Enterprises
HRC: Human–Robot Collaboration

## Footnotes

1 https://digitop.ac.uk/

2 https://aiprism.eu/

3 www.prolific.ac
